# Compliance with nutrition standards in Mexican schools and their effectiveness: a repeated cross-sectional study

**DOI:** 10.1186/s12889-018-6330-8

**Published:** 2018-12-27

**Authors:** Carolina Pérez-Ferrer, Tonatiuh Barrientos-Gutierrez, Juan A. Rivera-Dommarco, Francisco Javier Prado-Galbarro, Alejandra Jiménez-Aguilar, Carmen Morales-Ruán, Teresa Shamah-Levy

**Affiliations:** 1 Centro de Investigación en Salud Poblacional, Instituto Nacional de Salud Pública, Avenida Universidad 655, Santa María Ahuacatitlán, 62100 Cuernavaca, Morelos Mexico; 2 Centro de Investigación en Evaluación y Encuestas, Instituto Nacional de Salud Pública, Avenida Universidad 655, Santa María Ahuacatitlán, 62100 Cuernavaca, Morelos Mexico; 3 Dirección General, Instituto Nacional de Salud Pública, Avenida Universidad 655, Santa María Ahuacatitlán, 62100 Cuernavaca, Morelos Mexico

**Keywords:** Schools, Obesity, Nutrient-based standards, Policy, Mexico

## Abstract

**Background:**

Mexico approved mandatory nutrient-based standards for foods sold in schools in 2011. The aim of this study was to analyse the association between compliance with nutrition standards for foods sold in schools and children’s school snacks.

**Methods:**

Data came from three surveys representative of Mexican elementary schools in 13 states and their students (2012, 2013 and 2015); *n* = 645 children from *N* = 99 different schools. Information on foods sold in schools and snacks consumed by children was collected through direct observation. Compliance with the standards was defined as the proportion of foods sold in school which met nutrition criteria established by the standards. Snacks were classified as healthy if they contained at least one fruit or vegetable and had no sugar-sweetened beverages. Robust logistic regression models for cross-sectional and repeated surveys aggregated at the school-level were fitted to quantify the association between school compliance with standards and healthy snacks.

**Results:**

On average across waves 27% of foods sold complied with nutrition standards; 18% of children consumed a healthy snack. For snacks purchased in school, a 10% increase in school compliance with the standards was associated with a 32% increase in the odds of a healthy snack (OR = 1.32; 95%CI 1.09,1.61); no association was observed for snacks brought from home. The odds of a healthy snack increased over time in schools where compliance with the standards improved (OR = 3.89; 95%CI 1.47,10.31) but not in those where compliance remained constant or decreased.

**Conclusions:**

Only a small proportion of children are eating healthy snacks in school. School compliance with standards increases the likelihood of a healthy snack if it is bought at school. Our findings support better implementation of the standards and additional strategies to enhance the policy to achieve its aim of reducing childhood obesity.

**Electronic supplementary material:**

The online version of this article (10.1186/s12889-018-6330-8) contains supplementary material, which is available to authorized users.

## Background

Childhood obesity is a growing concern globally due to its rise in prevalence and its association with type 2 diabetes, dyslipidaemia, coronary artery diseases, and adult obesity [1, 2]. The World Health Organization (WHO) and other international organisations have proposed to intervene in the school food environment to reduce the prevalence of obesity in children [3, 4]. Food-based or nutrient-based standards for foods available in schools have been increasingly recognized as a key strategy to improve children’s diet [5]. Setting standards ensures a food environment that enables healthy choices and healthy preference learning early in life [5]. Evidence from the United States and United Kingdom show that nutrition standards for foods sold in schools have been effective at improving children’s diet during school hours [6–9].

In Mexico, one in three schoolchildren are obese, making childhood obesity one of the most important public health problems [10, 11]. Children in Mexico attend school in one of two shifts; morning from 8:30 to 13:30 or afternoon from 13:45 to 18:30. They do not eat a formal meal at school, they eat a snack, which may be brought from home or purchased in school [12]. In 2011, following WHO recommendations [4], the Mexican Ministries of Education and Health developed nutrition standards for foods and beverages sold in schools [13]. Standards were implemented in four stages to give the food industry time to reformulate their products. Nutrition criteria were revised at each stage, and limits for total fat, sugar and sodium were lowered (Table 1). The standards further recommend that snacks eaten in school should not exceed 250 kcal for elementary school children and include fruits, vegetables and plain water as opposed to sugar sweetened beverages [14]. Preliminary results have shown poor implementation of the standards and it is unclear whether they are having the intended effect over children’s diet and weight [15, 16]. There is an urgent need to strengthen the evidence base around the effectiveness of the standards in Mexico to either improve their implementation, or to change policy.

**Table 1:** Nutrition criteria according to each stage of the standards

Food categories	No.	Description	Stage 2	Stage 3	Stage 4
Prepared (fried and non-fried) foods	1	Portion per package	1	1	NA^a^
	2	Portion (kcal)	≤180	≤180	NA
	3	Proteins (% of calories)	≥10 (4.5 g)	≥10 (4.5 g)	NA
	4	Total fats (% of calories)	≤35	≤30	NA
	5	Saturated fats (%of calories)	≤15	≤10	NA
	6	Trans-fatty acids (g/portion)	≤0.5	≤0.5	NA
	7	Sodium (mg/portion)	≤230	≤220	NA
Milk	1	Portion per package	1	1	1
	2	Portion (ml)	≤250	≤250	≤250
	3	Calories per 100 g	≤50	≤50	≤50
	4	Total fats (g/100 g)	≤2.5	≤2.5	≤2.5
Solid yogurt	1	Portion per package	1	1	1
	2	Portion (g)	≤150	≤150	≤150
	3	Total fats (g/100 g)	≤2.5	≤2.5	≤2.5
	4	Total sugars (%)	NA	NA	≤30
Drinkable yogurt and fermented dairy products	1	Portion per package	1	1	1
	2	Portion (g)	≤250	≤200	≤200
	3	Total fats (g/100 g)	≤1.6	≤1.4	≤1.4
	4	Total sugars (%)	NA	NA	≤30
Fruit juices and vegetable juices	1	Portions per package	1	1	1
	2	Portion (ml)	≤200	≤125	≤125
	3	Calories per portion (kcal)	≤110	≤70	≤70
Sweetened fruit juices	1	Portions per package	1	1	1
	2	Portion (ml)	≤200	≤125	≤125
	3	Calories per portion (kcal)	≤110	≤70	≤70
Soy-based beverages	1	Portions per package	1	1	1
	2	Portion (ml)	≤200	≤125	≤125
	3	Calories per portion (kcal)	≤60	≤40	≤40
	4	Sodium (mg/100 ml)	≤110	≤105	≤105
	5	Total fats (g/100 ml)	≤2.5	≤2.5	≤2.5
	6	Saturated fats (%)	≤21% of total fats	≤21% of total fats	≤21% of total fats
Snacks	1	Portions per package	1	1	1
	2	Calories per portion (kcal)	≤130	≤130	≤130
	3	Total fats (% of calories)	≤40	≤35	≤35
	4	Saturated fats (%of calories)	≤25	≤15	≤15
	5	Trans-fatty acids (g/portion)	≤0.5	≤0.5	≤0.5
	6	Sodium (mg/portion)	≤200	≤180	≤180
	7	Total sugars (% of calories)	NA	NA	≤10
Cookies, snack cakes, candies and desertes	1	Portions per package	1	1	1
	2	Calories per portion (kcal)	≤130	≤130	≤130
	3	Total fats (% of calories)	≤40	≤35	≤35
	4	Saturated fats (%of calories)	≤20	≤15	≤15
	5	Trans-fatty acids (g/portion)	≤0.5	≤0.5	≤0.5
	6	Sodium (mg/portion)	≤200	≤180	≤180
	7	Total sugars (% of calories)	NA	NA	≤20
Nuts and dry legumes	1	Portions per package	1	1	1
	2	Calories per portion (kcal)	≤130	≤130	≤130
	3	Saturated fats (%of calories)	≤25	≤15	≤15
	4	Trans-fatty acids (g/portion)	≤0.5	≤0.5	≤0.5
	5	Sodium (mg/portion)	≤200	≤180	≤180
	6	Total sugars (% of calories	NA	NA	≤10

^a^Prepared foods (foods that are prepared from fresh ingredients in the school, for example sandwiches and tacos) are discouraged in stage 4 of the standards

This study aimed to investigate whether school compliance with the standards was associated with children bringing or purchasing a healthy snack to consume during school hours. We test two related hypotheses using data for stages 2, 3 and 4 (no data was available for stage one): 1) that better compliance with the standards would be directly associated with a healthy snack, in particular, if the snacks were purchased in the school, and 2) that the probability of a healthy snack would increase over time in schools in which compliance with the standards improved but not in those where compliance remained stable or worsened.

## Methods

### Data

**Fig. 1:**
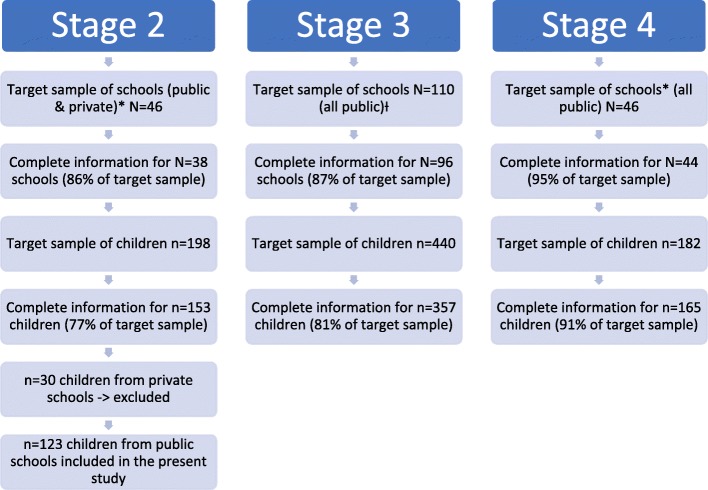
Sample selection procedures and response rates for each implementation stage data collection. *In stages 2 and 4 school level information was obtained from a larger sample of schools (*N* = 122 and *N* = 110 respectively). Only in a subsample of those were children observed. This diagram describes the sampling process starting from said subsample. Ɨ Funds for this stage allowed for a larger number of schools to be sampled following the same procedures as stages 2 and 4

Data came from three surveys representative of schools in 13 states of Mexico (out of 32) carried out at three implementation stages: June 2012, April 2013 and April 2015. The first survey included public and private elementary and high schools, while the last two included public elementary schools only. The present study focuses on morning and afternoon shifts of public elementary schools and their students. Sampling was done in two stages. First, clusters of schools were selected within strata (a combination of state, public/private and educational stage i.e. elementary or secondary), then children were randomly selected within schools (using a numbered list of all children in the target grades and selecting random numbers using random.org). A core group of schools were followed-up over time with a different sample of children drawn in each wave (*N* = 35 schools three time-points; *N* = 10 schools two time-points). On average four children per school were selected (min = 1, max = 5). Figure 1 illustrates the procedure followed in each stage, target and achieved samples with complete information at school and individual level for elementary schools. The total sample for the present study included 645 children from 99 different schools distributed as follows: 123 children from 38 schools in stage 2; 357 children from 96 schools in stage 3 and 165 children from 44 schools in stage 4. A complete case analysis was conducted.

Data collection in each survey consisted of a series of questionnaires and direct observations. Trained teams of interviewers (five-person teams) visited the schools and recorded the number of functioning drinking water sources. Then, the number of plates, packages or pieces of every single food and beverage available at the school food stores was recorded including their weight or volume. For packaged foods, interviewers recorded the number of portions contained in each package. For sampled children in each school, interviewers recorded basic demographic information, a list of foods consumed during school hours and whether the foods were brought from home or purchased in school. Interviewers were trained at the National Institute of Public Health in Cuernavaca. Questionnaires were tested for face validity in a pilot carried out in Cuernavaca.

### Study variables

#### Outcome variable

Healthy snack was a binary variable where one denotes a snack which contained at least one fruit or vegetable and did not contain sugar sweetened beverages. Information to construct this variable was obtained from direct observation of children’s lunchboxes and/or children’s purchases in school food stores. Sugar sweetened beverages included soda, industrialized juices, energy drinks and flavoured water which is prepared with added sugar and a small amount of fruit.

#### Exposure variables

School compliance with the standards was operationalized as the proportion of food items sold in school food stores that complied with the nutrition criteria in the different stages of the standards (Table 1). The numerator was the sum of foods and beverages sold in school that complied, and the denominator was the sum of all foods sold in the school. To calculate the numerator, the energy and macronutrient content of each food and beverage sold in schools and regulated by the standards was calculated using the nutrient composition tables compiled by the National Institute of Public Health [17]. Foods and beverages were grouped according to the food categories established in the guideline (see Table 1). Then according to the food group, we assessed compliance with the nutrition criteria. If the food item in question complied with n-1 (to allow for a few missing values) of the criteria established for its food group, we concluded that it complied with the guideline. Fruits, vegetables and plain water always complied.

Time was operationalized as stage and coded 0 for stage 2 (2012) 1 for stage 3 (2013) and 2 for stage 4 (2015).

#### Effect modifiers

Snack origin, whether the child’s snack was brought from home or purchased in the school (home = 0, school = 1).

Change in compliance, whether school’s compliance with the standards improved remained stable or declined over time, was constructed for the group of schools which were followed up for at least two timepoints. First, tertiles of the variable school compliance with the standards (described above) were created in each stage. This produced a measure of relative compliance useful for comparison across stages. Then, using the tertiles at the beginning and end of each school’s follow-up period, the variable change in compliance was created (0 = decline in compliance; 1 = stable; 2 = increase).

#### Covariates

Models were adjusted for individual and school-level confounders selected a priori. Individual level variables included: shift (1 = morning, 2 = afternoon), school grade (3rd, 4th, 5th 6th) and sex. School-level variables were availability of free drinking water, defined as at least one functioning water fountain or other communal water source in the school (1 = yes; 0 = no), municipal-level extreme poverty (continuous) and municipal-level education (continuous). Municipal-level extreme poverty was based on the proportion of households living in extreme poverty at the municipality where schools were located. Households in extreme poverty were defined as those having an income below the basic food basket value, calculated from the National Survey on Income and Expenditure 2014 [18]. Similarly, the proportion of the population over 24-years old at municipality level with completed high school or more was computed from the Intercensal Survey 2015 [19].

### *Statistical analysi*s

First, we tested hypothesis 1, that better school compliance with the standards would be directly associated with a healthy snack, in particular, if the snacks were purchased in the school. Data from the three survey waves were pooled and observations where part of the snack was purchased in school and part was brought from home were excluded. Before combining the data, the time variable was created to identify observations according to stage of the standards. We used logistic regression with adjustment for clustering at school level. The outcome variable was a healthy snack and the main exposure variable was school compliance with the standards. To assess effect modification, an interaction term between school compliance with standards and snack origin was included. Unadjusted and fully adjusted models were fitted. Predicted probabilities and probability differences for the effect of school compliance with standards on consumption of a healthy snack by whether the snack was brought from home or purchased in school were computed.

Hypothesis 2 was tested next, that the probability of a healthy snack would increase over time in schools in which compliance with the standards improved but not in those where compliance remained stable or worsened. Thus, we fitted a model for repeated surveys aggregated at the school level, keeping only schools that had information on compliance for at least two time-points for this analysis (*N* = 45). Further, observations where the snack or part of it was brought from home were excluded, since results for hypothesis 1 suggested that snacks brought from home were not affected by schools’ compliance with the standards. We used logistic regression with adjustment for clustering at school level. The outcome variable was a healthy snack and the main exposure variable was time. An interaction term was included between time and change in compliance. Unadjusted and a fully adjusted models were fitted, using the same covariates as the model used for hypothesis 1. Data management and all analyses were performed using Stata 12.

## Results

Schools selected for this study were distributed over 13 states of Mexico and 22 municipalities. On average 33% of adults over 25 years of age had completed high school education in the schools’ municipalities and 13% lived in extreme poverty (data not shown). On average across stages, 27% of foods sold in schools complied with the standards. Highest compliance was in stage 2 (less strict) at 42%. Availability of free drinking water decreased from 63% in stage 2 to 20% in stage 4. The mean age of children was 10 years old with girls making slightly over half of the population (Table 2). Only 18% of children across the three stages brought or purchased a healthy snack however, there was an improvement over time with more children bringing or buying a healthy snack in the more recent stages. 40% of children brought their snack from home, 33% purchased it at school and 26% both brought from home and purchased in school (Table 2). Area level poverty was negatively associated with a healthy snack if purchased at school while area level education was positively associated with a healthy snack if brought from home (Additional file 1: Table S1). Out of schools with follow-up in at least two time-points, 15 schools remained in the same tertile of compliance between their first and last follow-up period, 14 declined and 16 increased, approximately a third in each category.

**Table 2:** Descriptive characteristics of the children who participated in the study

	Stage 2	Stage 3	Stage 4	Total
N	123	357	165	645
Age (mean, sd)	11.0 (1.0)	9.9 (1.3)	9.7 (1.2)	10.1 (1.3)
Girls, N(%)	69 (56.1)	188 (52.7)	88 (53.3)	345 (53.5)
Grade, N (%)				
3rd	1 (0.8)	86 (24.1)	52 (31.5)	138 (21.4)
4th	44 (35.8)	112 (31.4)	46 (27.9)	202 (31.3)
5th	45 (36.6)	85 (23.8)	35 (21.2)	165 (25.6)
6th	33 (26.8)	74 (20.7)	32 (19.4)	139 (21.6)
Shift, N(%)				
Morning	95 (77.2)	245 (68.6)	128 (77.6)	468 (72.6)
Afternoon	28 (22.8)	112 (31.4)	37 (22.4)	177 (27.4)
Healthy snack	19 (15.5)	62 (17.4)	36 (21.8)	117 (18.1)
Snack origin N(%)				
Home	56 (45.5)	135 (37.8)	69 (41.8)	260 (40.3)
School	51 (41.5)	109 (30.5)	57 (34.6)	217 (33.6)
Home and school	16 (13.0)	113 (31.7)	39 (23.6)	168 (26.1)

There was effect modification in the association between compliance with the standards and healthy snack by the origin of the snack (effect modification *p* = 0.05). If the snack was purchased in school, a 10% increase in school compliance with the standards was associated with a 32% increase in the odds of a healthy snack in fully adjusted models (OR = 1.32 95%CI 1.09,1.61) whereas, if the snack was brought from home, school compliance with the standards was not associated with children consuming a healthy snack (OR = 1.01 95%CI 0.81,1.26) (Table 3 and Additional file 1: Table S2 for the full model). The difference in the probability of a healthy snack if the snack was purchased in school minus the probability of a healthy snack if the snack was brought from home increased as school compliance with standards increased.

**Table 3:** Odds ratio (95% CI) of a healthy snack for a 10% increase in school compliance with standards, stratified by snack origin (*n* = 477)

	Unadjusted model		Adjusted model^a^	
	OR (95%CI)	Effect mod. p ^b^	OR (95%CI)	Effect mod. p ^b^
Snack from home	0.97 (0.77,1.22)	0.10	1.01 (0.81,1.26)	0.05
Snack from school	1.21 (1.02,1.44)		1.32 (1.09,1.61)	

^a^Adjusted for time, sex, grade, shift, free drinking water, area level education and area level extreme poverty ^b^ For the null hypothesis that the association between school compliance to standards and healthy snack is the same regardless of whether the snack was brought from home or purchased in school

Table 4 shows the odds ratio of consumption of a healthy snack for a one period increase in time and stratified by change in compliance with the standards. In schools where compliance with the standards increased over time, the adjusted odds of a healthy snack increased for a one-unit increase in time (OR = 3.89 95% CI 1.47,10.31) whereas in schools where compliance declined or remained stable, the odds of a healthy snack among children had a declining trend over time (OR = 0.57 95% CI 0.19,1.70 and OR = 0.58 95% CI 0.25,1.37 respectively; effect modification *p* = 0.006). To see the measures of effect of all covariates see Additional file 1: Table S3.

**Table 4:** Odds ratio of consumption of a healthy snack for a one period increase in time stratified by change in compliance to the standards (*n* = 154)

	Unadjusted model		Adjusted model^a^	
	OR (95%CI)	Effect mod. p^b^	OR (95%CI)	Effect mod. p^b^
Compliance decreases	0.77 (0.37,1.62)	0.2579	0.57 (0.19,1.70)	0.006
Compliance stable	0.63 (0.32,1.22)		0.58 (0.25,1.37)	
Compliance increases	2.33 (0.91,5.98)		3.89 (1.47,10.31)	

^a^Adjustments: sex, grade, shift, area level extreme poverty, area level education and free drinking water. ^b^For the null hypothesis that the odds of a healthy snack over time is the same regardless of whether school’s compliance with the standards increases, is stable or decreases

## Discussion

Our study is the first to examine the association between compliance with the Mexican school food standards and snacks consumed by children during school hours. Our findings show that better compliance with the standards is associated with an increased probability of consuming a healthy snack if the snacks were purchased in the school but not if they were brought from home. Further, our study finds that the probability of consuming a healthy snack increased over time in schools in which compliance with the standards improved but not in others.

In the Mexican context, where childhood obesity prevalence is very high, the findings of this study are highly relevant and encouraging for public health policy. By exploiting heterogeneity in school compliance with the standards we were able to identify the policy’s effectiveness over snacks purchased in school. This finding is consistent with the recommendation to implement policy actions that improve the school environment by offering healthy choices to children [4]. It is also consistent with the conclusions of a systematic review which examined the effect of regulations on the sale of foods and beverages outside of the school meal programs in the United States on children’s diet during school hours [6]. In most studies included in the review, this type of regulation was associated with changes in consumption and/or availability of regulated foods in the expected direction [6]. Standards for foods available in schools, such as the Mexican standards, are sustainable over time compared to other school-based interventions like provision of fruits and vegetables which needs constant financing. Further, because this intervention requires little agency from individuals as it modifies the environment in which food choices are made, it is expected to show equal or greater benefit for lower socioeconomic groups [20]. This is very important in countries undergoing the nutrition transition, like Mexico, where obesity prevalence is shifting to socially disadvantaged populations [21].

In addition to directly affecting food products sold in schools, the standards set out recommendations for healthy snacks prepared at home and for communication with parents regarding healthy eating [13, 14]. There is evidence from the UK that food brought from home can be improved through intervention [22]. Therefore, it was plausible to expect that snacks brought from home would be healthier in schools with higher compliance with the standards. However, this study found that school compliance with the standards was not associated with healthy snacks brought from home. This may be due to children’s pre-existing preferences for unhealthy foods learned in the pre-school years which undermine policies in school [23, 24]. There is evidence from the United States that children compensate by bringing the restricted foods and drinks from home or consume more food outside of school [25]. There may be other factors which influence snacks brought from home which are beyond the scope of the standards, for example the household’s socioeconomic position or the parent’s education.

This study also found that school compliance with the standards was in general very poor and very few children brought or purchased a healthy snack to consume during school hours. Free water availability decreased over time and schools were not able to keep up and comply with the changes in the standards. This is consistent with two other studies carried out in Mexico recently [15, 16]. The first described the foods available for sale in schools during the first two stages of implementation and found that sale of energy-dense nutrient-poor foods persisted regardless of the standards, and availability of healthier options continued to be low [16]. The second study described children’s food consumption during school hours and found that after implementation of the standards, children were still consuming significantly more energy, sugar and fat than recommended [15]. Water fountains were installed in schools around the time that the standards were implemented. However, no budget was set aside for maintenance (e.g. for changing filters) which is reflected in the decline of free drinking water availability over time. It is clear from our study’s findings that there is great scope for improvement.

The design of the Mexican standards was challenging and there were many setbacks. Still, Mexico managed to approve the policy, a great win for the public health community. There are areas of opportunity to enhance the effectiveness of the standards based on the results of this study. In order to improve snacks brought from home, some suggestions would be to increase parental involvement and apply nutrition standards to other venues and purchasing channels near the schools since the number of food vendors around a school has been found to be directly associated with children’s eating habits and body mass index [26–28]. To improve implementation of the standards within schools, actions such as supplying healthy substitutes for popular foods and drinks, further engaging with staff involved in food delivery and teachers [5], ensuring provision of free drinking water, regulating advertising of unhealthy foods in schools and around them and not penalizing freshly prepared foods that are prepared hygienically and form part of the traditional Mexican diet are some of the options. Current standards encourage consumption of fruit and vegetables, wholegrain cereals, seeds and nuts and allow consumption of smaller portions of ultra-processed foods which meet nutrition criteria (only on Friday as of 2015); however, they penalize freshly prepared dishes. This may inadvertently teach children that ultra-processed foods are desirable and better than freshly prepared traditional dishes. There is increasing scientific evidence linking ultra-processed food products, as a group, with unhealthy dietary practices, obesity and non-communicable diseases [29].

Our study has several strengths. It includes data from three comparable surveys spread across a period of four years. Surveys included schools from 13 states of the country and a good proportion of them were followed up over time. This allowed for a more robust analysis in which we were able to show differential changes in the odds of a healthy snack according to changes in compliance with the standards over time. This study used objectively measured exposure and outcome variables thus reducing information bias. Further, we believe our findings to have high external validity since they arise from real world experience of policy implementation. The sample was representative of the population of elementary level children studying at public schools in selected states.

This study also has some limitations. It was beyond the scope of our study to formally evaluate the policy (standards) for two reasons. The first is that no pre-policy measurements exist on the offer of foods in schools and food consumption of children in schools in a nationally representative survey. The second, is that because this policy was implemented at national level in 2012, there was no control group of schools available. Nevertheless, we believe our findings strengthen the evidence base around the standards and justify the continuation of the policy and its better implementation. We show that if schools offer healthier options, children consume healthier snacks. Another limitation is the cross-sectional nature of the data. We cannot rule out reverse causality, that the demand for healthier snacks from the children leads to schools offering healthier foods. Further, this study only evaluates food consumed by children during school hours. We are unable to comment on whether the standards have had an impact on overall dietary consumption and/or nutrition status. The surveys did not include an assessment of children’s overall diet neither did they include anthropometric measurements. It was outside the scope of this study to investigate potential determinants of school compliance with the standards and/or factors affecting student’s preference for unhealthy snacks. Future research could explore these two interesting topics.

## Conclusion

In conclusion, our study found that school compliance with nutrition standards for foods sold in schools was directly associated with children purchasing a healthy snack during school hours and not associated with healthy snacks if brought from home. The odds of a healthy snack increased over time in schools in which compliance with the standards improved but not in schools where it remained stable or declined. Further, the study found that school compliance with the standards and the proportion of children consuming healthy snacks were both very low leaving much room for improvement. Our findings support doubling efforts to better implement the standards and call for additional strategies to enhance the policy so that it can achieve its aim of reducing childhood obesity.

## Additional file

Additional file 1: **Table S1.** Bivariate associations with healthy snack. **Table S2.** Odds ratio (95% CI) of a healthy snack for a 10% increase in school compliance with standards, including measures of effect for all covariates*. **Table S3.** Odds ratio of consumption of a healthy snack for a one period increase in time, including measures of effect for all covariates. (DOCX 18 kb)

## Abbreviations

CI: Confidence interval
OR: Odds ratio
WHO: World Health Organization
