# Supplementary material for: Compliance with nutrition standards in Mexican schools and their effectiveness: a repeated cross-sectional study
Source: BMC Public Health. 2018 Dec 27;18:1411. doi: 10.1186/s12889-018-6330-8 (PMC6307217; doi:10.1186/s12889-018-6330-8)
Supplement: Supplementary file 1 — Table S1. Bivariate associations with healthy snack. Table S2. Odds ratio (95% CI) of a healthy snack for a 10% increase in school compliance with standards, including measures of effect for all covariates*. Table S3. Odds ratio of consumption of a healthy snack for a one period increase in time, including measures of effect for all covariates. (DOCX 18 kb) [file 12889_2018_6330_MOESM1_ESM.docx]

Supplementary Table S1: Bivariate associations with healthy snack

|  |  | **Snack purchased at school** | | | **Snack brought from home** | | |
| --- | --- | --- | --- | --- | --- | --- | --- |
|  |  | **Healthy*** | **Not healthy*** | **p^Ɨ^** | **Healthy*** | **Not healthy*** | **p^Ɨ^** |
| Sex | Boys | 45.7 | 47.8 | 0.821 | 54.1 | 47.5 | 0.462 |
|  | Girls | 54.3 | 52.2 |  | 46.0 | 52.5 |  |
| Grade | 3rd | 11.4 | 16.5 | 0.810 | 18.9 | 24.7 | 0.616 |
|  | 4th | 31.4 | 24.7 |  | 37.8 | 34.1 |  |
|  | 5th | 31.4 | 27.5 |  | 21.6 | 26.5 |  |
|  | 6th | 25.7 | 30.8 |  | 21.6 | 14.8 |  |
| Shift | morning | 71.4 | 64.3 | 0.416 | 62.2 | 82.5 | **0.004** |
|  | afternoon | 28.6 | 35.7 |  | 37.8 | 17.5 |  |
| Stage | 2nd | 22.9 | 23.6 | 0.944 | 13.5 | 22.9 | **0.092** |
|  | 3rd | 48.6 | 50.6 |  | 46.0 | 52.9 |  |
|  | 4th | 28.6 | 25.8 |  | 40.5 | 24.2 |  |
| Free drinking water, mean | | 40.0 | 30.8 | 0.287 | 29.7 | 32.7 | 0.719 |
| Areal level education, mean | | 35.1 | 33.2 | 0.356 | 38.8 | 32.7 | **0.001** |
| Area level extreme poverty, mean | | 11.9 | 14.9 | **0.008** | 13.6 | 12.8 | 0.473 |
| Compliance with standards, mean | | 30.3 | 22.1 | **0.025** | 24.1 | 23.0 | 0.755 |

*Proportions presented when mean not stated. **^Ɨ^** For the null hypothesis that the proportion or mean of the variable (i.e. sex, grade, free drinking water) is the same among children with and without healthy snack. Hypothesis tests for differences in proportions and means were carried out with chi2 and ttest respectively.

Supplementary Table S2: Odds ratio (95% CI) of a healthy snack for a 10% increase in school compliance with standards, including measures of effect for all covariates*

|  | **Unadjusted model** | **Adjusted model** |
| --- | --- | --- |
|  | **OR (95%CI)** | **OR (95%CI)** |
| **Compliance with standards#home** | 0.97 (0.77,1.22) | 1.01 (0.81,1.26) |
| **Compliance with standards#school** | **1.21 (1.02,1.44)** | **1.32 (1.09,1.61)** |
|  |  |  |
| Time (continuous) |  | **1.85 (1.24,2.73)** |
| Sex (ref=boys) |  | 0.90 (0.48,1.69) |
| Grade (continuous) |  | 1.12 (0.88,1.44) |
| Shift (ref=morning) |  | 1.88 (0.95,3.69) |
| Free drinking water (ref=no) |  | 1.13 (0.55,2.31) |
| Area education (continuous) |  | **1.04 (1.01,1.07)** |
| Area poverty (continuous) |  | - 1. 0.93,1.02) |

*Time (0=stage 2, 1=stage 3, 2=stage 4), sex (1=boy, 2=girl), school grade (1=3^rd^, 2=4^th^, 3=5^th^ , 4=6^th^), shift (1=morning, 2=afternoon), availability of free drinking water (1=yes, 0=no), municipal-level education (continuous proportion) and municipal-level extreme poverty (continuous proportion).

Supplementary Table S3: Odds ratio of consumption of a healthy snack for a one period increase in time, including measures of effect for all covariates

|  | **Unadjusted model** | **Adjusted model** |
| --- | --- | --- |
|  | **OR (95%CI)** | **OR (95%CI)** |
| **Time#Compliance decreases** | 0.77 (0.37,1.62) | 0.57 (0.19,1.70) |
| **Time#Compliance stable** | 0.63 (0.32,1.22) | 0.58 (0.25,1.37) |
| **Time#Compliance increases** | 2.33 (0.91,5.98) | **3.89 (1.47,10.31)** |
|  |  |  |
| Sex (ref=boys) |  | 1.71 (0.57,5.09) |
| Grade (continuous) |  | 0.89 (0.61,1.32) |
| Shift (ref=morning) |  | **0.22 (0.06,0.86)** |
| Free drinking water (ref=no) |  | 1.82 (0.46,7.15) |
| Area education (continuous) |  | 1.03 (0.98,1.07) |
| Area poverty (continuous) |  | 0.88 (0.79,0.98) |

*Sex (1=boy, 2=girl), school grade (1=3^rd^, 2=4^th^, 3=5^th^ , 4=6^th^), shift (1=morning, 2=afternoon), availability of free drinking water (1=yes, 0=no), municipal-level education (continuous proportion) and municipal-level extreme poverty (continuous proportion).
